# Couple-Based Communication Interventions for Cancer Patient–Spousal Caregiver Dyads’ Psychosocial Adaptation to Cancer: A Systematic Review

**DOI:** 10.3390/healthcare11020236

**Published:** 2023-01-12

**Authors:** Junrui Zhou, Xuan Chen, Zhiming Wang, Qiuping Li

**Affiliations:** Wuxi School of Medicine, Jiangnan University, Wuxi 214122, China

**Keywords:** communication, intervention, program, cancer, couple, patient, spousal caregiver, adaptation

## Abstract

(1) Background: Effective communication among couples in which one has been diagnosed with cancer is critical to improve their psychosocial adaptation to cancer. The objective of this review was to explore the characteristics and measurement outcomes of existing couple-based communication interventions in the cancer context. (2) Methods: Eight electronic databases were searched from database initiation to August 2022 to identify eligible articles. Hand searching was also performed on the included articles’ reference lists and authors. (3) Results: A total of 14 intervention studies were eligible to be included in this review. Cancer couples with distress or communication problems before intervention were more likely to benefit from the couple-based communication interventions. Positive outcomes were reported, including an improvement in relationship functioning (including mutual communication, intimacy, and relationship satisfaction) and individual functioning (including a decline of anxiety, depression and cancer-related concerns, and an increase in psychological adjustment and quality of life). (4) Conclusions: These findings supported the importance of improving mutual communication behaviors to promote cancer patient–spousal caregiver dyads’ psychosocial adaptation to cancer. While most included studies were conducted in western countries and the sample size was relatively small, more research is warranted to develop more efficacious couple-based communication interventions.

## 1. Introduction

According to updated global cancer statistics, approximately 19.3 million new cancer cases and an estimated 9.9 million cancer deaths occurred in 2020 for 36 types of cancers in 185 countries [1]. Furthermore, the global incidence of cancer is expected to increase to 28.4 million in 2040 [1]. According to an assessment by the World Health Organization (WHO), cancer was the first or second leading cause of death for the majority of countries in the world during the period from 2000 to 2019 [2]. The high incidence and mortality of cancer suggest that cancer-related issues are still a primary global disease burden. For those cancer patients who are married or in a committed relationship, patients and their spousal caregivers (SCs) generally experienced mutually related adaptation outcomes, such as self-efficacy, mental health, role adjustment, marital satisfaction, and quality of life (QOL) [3]. A study conducted by Lin et al., which focused on colorectal cancer patients (CPs) and their SCs, also found that CPs were positively related to their SCs, and vice versa [4]. Given the reciprocal influence among CPs and SCs, a growing number of researchers have advised to view cancer from a relational or dyadic perspective [5,6,7]. Kayser et al. [8] regard cancer as a “we-disease”, which distinguished ownership of cancer. “We-disease” means that CPs and their SCs regard cancer as theirs and take efforts to communally cope with cancer-related stress as a couple [8]. Badr et al. [9] explored the effect of pronoun use during the natural communication context on a couple’s psychosocial adaptation to head and neck cancer. They reported that more usage of first-person plural pronouns (e.g., we and ours) was related to a reduction in couples’ distress, while more usage of first-person singular pronouns (e.g., I and mine) was related to couples’ lower levels of marital satisfaction, which further supported the view that it is necessary and important to view CPs and SCs as CP–SC dyads. 

Mutual communication plays a critical role in the psychosocial adaptation to cancer for CP–SC dyads. A review, which explored cancer couples’ mutual impacts, reported that communication may be the fundamental element of the three concepts (which included “communication”, “reciprocal influence”, and “caregiver–patient congruence”), and better communication may facilitate an improvement in the other two concepts [3]. In addition, another review explored the impact paths of communication (among CPs and their family caregivers) on the adjustment to cancer based on 116 relevant studies [10]. Theoretical and empirical evidence supported the view that communication could impact the adjustment to cancer through at least three paths [10]. First, open communication could facilitate better coping through discussing and implementing methods of problem-solving, and further improve the adjustment to cancer. Second, as a kind of interactive coping mechanism (e.g., seeking support from spouses, discussing feelings and making new future plans with partners), communication could directly impact the adjustment to cancer. Third, cycling back and forth between communication patterns and coping strategies could jointly impact further adjustments to cancer. Except for impact paths, different communication patterns would have a different influence on CP–SC dyads’ psychosocial adaptations to cancer. Based on previous theories and empiric studies, Manne et al. [11] proposed the Relationship Intimacy Model (RIM), which views cancer from a relational perspective and regards the marital relationship as a resource for dyadic couples coping with cancer. RIM is a dyadic-level theory which aims to promote CP–SP dyads’ psychosocial adaptation to cancer through strengthening relationship-enhancing behaviors (including reciprocal self-disclosure, partner responsiveness, and relationship engagement) and decreasing relationship-compromising behaviors (including avoidance, criticism, and pressure-withdraw). Studies reported that mutual constructive communication between CPs and SCs was associated with greater relationship satisfaction (RS) and reduced psychological distress and depression, whereas mutual avoidance, critical communication and pressure-withdraw were related to lower RS, greater psychological distress and depression, and reduced well-being and psychological adjustment [3,12]. In addition, RIM also hypothesizes that relationship-enhancing and relationship-compromising behaviors could indirectly impact dyads’ psychosocial adaptation to cancer by changing their intimacy [11]. Studies which focused on prostate CP–SC dyads reported that mutual constructive communication reduced psychological distress by promoting intimacy [13], while mutual avoidance, holding back and patient demand–partner withdrawal decreased RS and/or increased psychological distress by reducing intimacy [14]. Based on the aforementioned evidence, communication could impact CP–SC dyads’ psychosocial adaptation to cancer in different ways. In this review, the psychosocial adaptation is categorized into two aspects, including relationship and individual functioning. Relationship functioning involves mutual communication, intimacy and RS, while individual functioning includes negative functioning (e.g., psychological distress and depression) and positive functioning (e.g., well-being and psychological adjustment). 

With accumulating attention to relational perspective in the cancer context, a burgeoning number of couple-based interventions have emerged [15,16]. Most of these interventions comprised communication skill training components [17]. Couple-based communication plays an important role in helping couples understand cancer experiences, engage in social support, discuss role and responsibility changes and promote communal coping with cancer [17,18]. A meta-analysis reported that couple-based interventions for improving CP–SC dyads’ adaptation to cancer had small but beneficial effects (Cohen’s d = 0.25–0.31 for CPs, and Cohen’s d = 0.21–0.24 for SCs) [19]. The most common reasons for these small effects were different or absent theoretical formats, varied intervention approaches and diverse measurements of outcomes [19]. Luo [20] provided multiple topics and comprehensive components for colorectal CP–SC dyads in her intervention study, and suggested that more focused content may make interventions more effective (e.g., focusing on couples’ communication skills). At present, a systematic review assessing the effect of couple-based communication interventions on CP–SC dyads’ psychosocial adaptation to cancer is lacking. In addition, a summary of the characteristics of the existing interventions is still needed to refine couple-based communication interventions for CP–SC dyads. Therefore, this review aims to (a) summarize the approach and contents of couple-based communication interventions in the cancer context, (b) explore these interventions’ feasibility and acceptability, (c) review their impact on couples’ relationship and/or individual functioning and (d) identify future research directions. 

## 2. Methods

### 2.1. Search Methods for Eligible Articles

**Figure 1:**
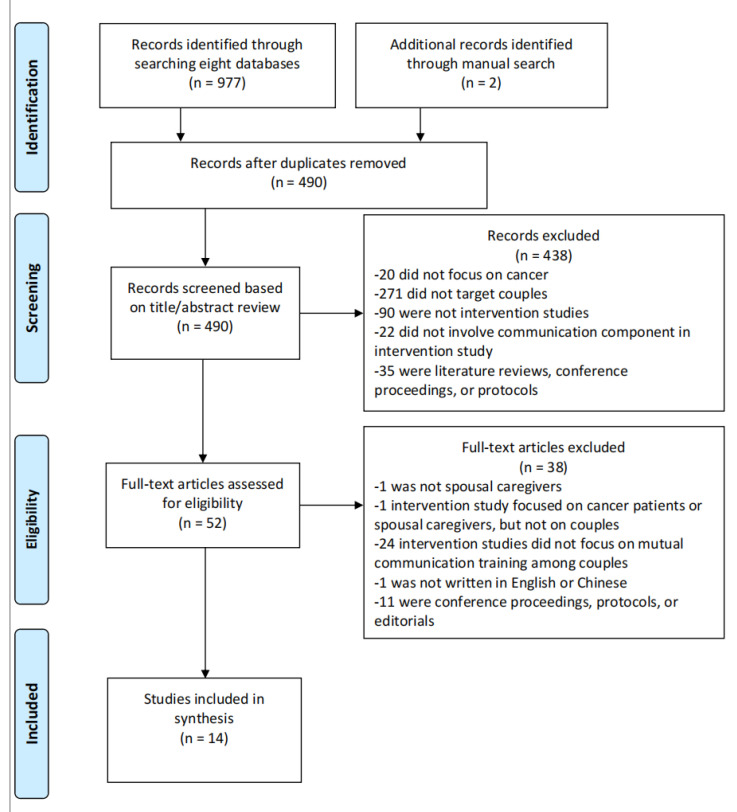
The flow diagram on identifying eligible articles in the literature.

This review was performed in accordance with the PRISMA (Preferred Reporting Items for Systematic Reviews and Meta-Analyses) guidelines. A systematic search was performed to find all eligible studies which were published in English or Chinese. Six English databases (including CINAHL, Cochrane, Embase, Medline, PsyINFO, PubMed) and two databases (including CNKI and Wanfang Data) from China were searched from database initiation to August 2022. For the six English databases, the following key terms and their synonyms, which were limited to the title or abstract, were used: “cancer” or “tumor” or “oncology” AND “couple” or “spouse” or “partner” AND “communication” or “conservation” or “emotional disclosure” AND “program” or “training” or “education” or “intervention”. Regarding the two Chinese databases, we used “肿瘤” (tumor) or “癌症” (cancer) AND “夫妻” (couple) or “配偶” (spouse) or “伴侣” (partner) AND “沟通” (communication) or “交流” (conversation) AND “干预” (intervention) or “健康教育” (healthy education) as key words to search for eligible studies. For the purpose of finding as many relevant articles as possible, the outcome measures (e.g., “relationship satisfaction” or “psychological distress”) were not included in the search terms, but they were considered during the selection process according to the inclusion and exclusion criteria. A manual search of reference lists of included studies and the relevant authors was also conducted to find additional eligible studies. Figure 1 shows a flowchart of the searching and selection process.

### 2.2. Selection Criteria for Identifying Articles

The inclusion criteria were as follows: (1) target population of studies was couples in which one had a diagnosis of any type of cancer; (2) interventions specially focused on couple-based communication components; (3) both the patients’ and spouses’ relationship and/or individual functioning were measured; (4) studies were completely published in peer-reviewed journals in English or Chinese.

The exclusion criteria were as follows: (1) except for SCs, informal caregivers also included other family members or friends; (2) interventions focused on CPs or SCs, but not CP–SC dyads; (3) interventions paid attention to other components (e.g., mindfulness training), instead of focusing on couple-based communication skill training; (4) studies were protocols, reviews, theses, conference proceedings or editorials.

### 2.3. Data Extraction and Synthesis

Two tables were used to fully extract and synthesize important information in the included studies. In Table 1, we primarily summarized the characteristics of the intervention articles, including the author, published year, country where the study was conducted, study aims, study design, characteristics of cancer and participants, theoretical framework, intervention component and dosage and delivery format. In Table 2, we synthesized the included studies’ measurement tools and intervention outcomes.

### 2.4. Quality Assessment

We selected and used the Effective Public Health Practice Project (EPHPP) to assess the QR of included articles in this review. The EPHPP is a universal and comprehensive scale to examine bias in aspects of selection, design, confounders, data collection methods and withdrawals and dropouts for a series of study designs [21]. In addition, the EPHPP is regarded as a useful tool for systematic reviews that focus on intervention studies [22]. Table 3 provides detailed information of this assessment tool and the QR of included studies.

**Table 1:** Characteristics of included studies.

Author (Year) Country [Reference]	Study Aims	Study Design	Cancer Diagnosis (Stages); Characteristic and No. of Participants	Theoretical Framework	Intervention Content and Dosage	Delivery Format (Who and How)
Fergus et al. (2014) Canada [23]	-To assess feasibility and acceptability of the program; -To explore improved areas of participants after completing the program.	Single group study	Breast cancer (stages 0–3); All eligible participants regardless of level of relationship and individual functioning before intervention; **Intervention Group (IG):** 16 dyads.	-Developmental model of couple adaptation to illness (Rolland); -Theory of healthy relationship functioning (Gottman); -Models of dyadic coping (Bodenmann).	**Reciprocal self-disclosure:** (1) disclosure of own and partner’s preference and experience through answering a series of topics (including cancer). **Partner responsiveness:** (2) review and discuss own turning-toward and turning-away behaviors. **Relationship engagement:** (1) communicate individual and relationship strengths; (2) think metaphorically about cancer and create a visual representation of cancer to fortify sense of “we-ness”; (3) watch video to learn sharing self-concerns and understand partner’s perspective; (4) co-construct a relationship line about couple’s pivotal events/periods and identify new life goals; (5) communally communicate with facilitators through the private website’s ‘Dialogue Room’ discussion board; six modules (once weekly) within eight weeks.	-Mental health professional; -Online and telephone.
Fergus et al. (2022) Canada [24]	-To explore effect of the program on couple’s relationship and individual functioning.	Randomized controlled trial (RCT)	Breast cancer (stages 0–3); All eligible participants, regardless of level of relationship and individual functioning before intervention; **IG:** 31 dyads; **Control group (CG):** 36 dyads.	-Systemic constructivist metatheory; -Developmental model of couple adaptation to illness (Rolland); -Theory of healthy relationship functioning (Gottman).	**Reciprocal self-disclosure:** (1) the same as the one (Fergus et al. [23]). **Partner responsiveness:** (1) the same as the one (Fergus et al. [23]). **Relationship engagement:** (1) discuss each physically pleasurable shared time and practice sensate focus exercise; (2) remaining contents were the same as the one (Fergus et al. [23]); six modules (once weekly) within eight weeks.	-Mental health professional; -Online and telephone.
Fergus et al. (2022) Canada [25]	-To assess acceptability of the couplelinks online program; -To explore gains of participants.	Single group study	Breast cancer (stages 0–3); All eligible participants regardless of relationship and individual functioning before intervention; **IG:** 30 dyads	-Systemic constructivist metatheory; -Developmental model of couple adaptation to illness (Rolland); -Theory of healthy relationship functioning (Gottman).	Intervention contents and dosage were the same as the one (Fergus et al. [24]).	-Mental health professional; -Online and telephone.
Gremore et al. (2020) USA [26]	-To test feasibility and acceptability of a couple-based supportive communication (CSC) intervention; -To explore efficacy of CSC.	RCT	Head and neck cancer (stages 1–4a); All eligible participants regardless of relationship and individual functioning before intervention; **IG:** 10 dyads; **CG:** 10 dyads.	-Social-cognitive processing theory; -Relationship intimacy model (RIM).	**Reciprocal self-disclosure:** (1) express own vulnerable feelings. **Partner responsiveness:** (1) patiently listen to partner’s disclosure and response with empathy and validation. **Relationship engagement:** (1) highlight individual and relationship strengths; (2) communicate supportively to understand problems rather than transitioning too quickly to problem-solving communication; (3) identify frequency of received and preferred support through completing support in intimate relationship rating scale (SIRRS); (4) review supportive communication skills and discuss transition to survivorship phase. **Relationship-compromising behaviors:** (1) block negative behaviors (e.g., invalidation); four sessions (each 75 min).	-Psychologist; -In person or video conference.
Manne et al. (2011) USA [27]	-To examine effects of the intimacy-enhancing therapy (IET) intervention; -To evaluate the role of baseline outcome variables in effects of IET on couples’ relationship and psychological functioning.	RCT	Prostate cancer (stages 1–2); All eligible participants regardless of level of relationship and individual functioning before intervention; -**IG:** 37 dyads; -**CG:** 34 dyads.	-RIM.	**Reciprocal self-disclosure:** (1) improve couple’s ability to comfortably share their cancer-related thoughts and feelings. **Partner responsiveness:** (1) improve mutual understanding. **Relationship engagement:** (1) improve constructive communication about cancer-related concerns, mutual support, and emotional intimacy; (2) complete in-session skill practice and home assignments; five sessions (each 90 min) within 8 weeks.	-Therapists; -In person.
Mowll et al. (2015) Australia [28]	-To explore feasibility and acceptability of patients dignity inventory couple interview (PDI-CI) intervention; -To determine effect of PDI-CI on couples.	Single group study	Cancer (advanced); All eligible participants regardless of level of relationship and individual functioning before intervention; **IG:** 9 dyads.	Not reported	**Reciprocal self-disclosure:** (1) individually complete PDI based on their own perception of patient’s situation. **Relationship engagement:** (1) review results and identify concurrence and discordance with the psychologist, facilitate matched communication; one session (60 min).	-Psychologist; -In person.
Porter et al. (2009) USA [29]	-To explore efficacy of partner-assisted emotional disclosure intervention comparing with providing cancer-related information.	RCT	Gastrointestinal (GI) cancer (stages 2–4); All eligible participants regardless of relationship and individual functioning before intervention; **IG:** 65 dyads; **CG:** 65 dyads.	Not reported	**Unidirectional self-disclosure:** (1) patients list their cancer-related concerns and disclose their own cancer-related events and feelings in as much detail as possible; **Unidirectional partner responsiveness:** (1) partners reflectively listen to patients’ disclosure, try to understand cancer experience in patients’ place, and avoid problem-solving, reassurance or advice giving; (2) identify helpful partner responses. **Relationship-compromising behaviors:** (1) identify unhelpful partner responses; four sessions (the first 75 min, the last three 45 min each) within four to eight weeks.	-Social worker or psychologist; -In person.
Porter et al. (2012) USA [30]	-To explore efficacy of partner-assisted emotional disclosure intervention comparing with providing cancer-related information; -To determine process variables that may influence the intervention effects.	RCT	GI cancer (stages 2–4); All eligible participants regardless of level of relationship and individual functioning before intervention; **IG:** 65 dyads; **CG:** 65 dyads.	Not reported	Intervention contents and dosage were the same as the one (Porter et al. [29]).	-Social workers or psychologists; -In person.
Porter et al. (2012) USA [31]	-To explore couples’ experience in partner-assisted emotional disclosure intervention; -To determine variables associated with ratings of couples’ communication.	Single group study	GI cancer (stages 2–4); All eligible participants regardless of level relationship and individual functioning before intervention; **IG:** 47 dyads.	Not reported	Intervention contents and dosage were the same as the one (Porter et al. [29]) above.	-Social workers or psychologists; -In person.
Shields et al. (2004) USA [32]	-To assess feasibility of the group intervention; -To explore efficacy of the group intervention.	Three groups study	Breast cancer (all stages); All eligible participants regardless of level of relationship and individual functioning before intervention; **2-session:** 12 dyads; **1-session:** 22 dyads; **CG:** 11 dyads.	Not reported	**Reciprocal self-disclosure:** (1) express cancer-related experience, cognition and emotions; (2) patients and partners express their own bridges and barriers to communicate with the other separately. **Partner responsiveness:** (1) listen supportively to partner’s disclosure and gain new insights into their experience. **Relationship engagement:** (1) discuss bridges and barriers as a couple; (2) co-construct a relationship line about up or down times, plan for their life post-cancer and share and discuss the relationship line with other couples; 2-session group intervention: two sessions (each four hours); 1-session group intervention: one session (four hours) which was eliminated part of “bridges and barriers”.	-Not reported; -Offline group intervention.
Manne et al. (2019) USA [33]	-To compare the impact of IET, a general health and wellness (GHW) intervention, and Usual Care (UC) on couples’ relationship and psychological functioning.	RCT	Prostate cancer (stages 1–3); Ones who had distress before intervention; **IG:** 80 dyads **CG:** GHW: 76 dyads; UC: 81 dyads.	-RIM.	**Reciprocal self-disclosure:** (1) disclosure of cancer experience and sources of distress and create a list of cancer concerns; (2) express support needs. **Partner responsiveness:** (1) respond with empathy and validation. **Relationship engagement:** (1) learn problem-solving model; (2) practice speaking, listening and problem-solving skill with a cancer concern during in-session exercise and home assignment, and practice sensate focus exercise at home; (3) review skills and challenges, and troubleshoot future issues; six sessions (each 90 min) and one booster call (30–45 min).	-Psychologist or social worker; -In person and telephone.
Porter et al. (2017) USA [34]	-To determine the feasibility and acceptability of the couples communication skills training (CCST) intervention; -To explore effects of CCST compared with an educational intervention (HLI).	RCT	GI cancer (stage 4); Ones who presented holding back pattern before intervention; **IG:** 15 dyads; **CG:** 17 dyads.	Not reported	**Reciprocal self-disclosure:** (1) share thoughts and feelings about a series of cancer-related topics. **Partner responsiveness:** (1) accept and affirm partners’ feelings and perspectives. **Relationship engagement:** (1) try to make a cancer-related decision through communication skills; (2) review progress during treatment and identify future issues related to communicating about cancer; six sessions (each 60 min).	-Social workers; -Video conference.
Porter et al. (2018) USA [35]	-To determine themes discussed by couples during CCST intervention.	Single group study	GI cancer (advanced); Ones who presented holding back pattern before intervention; **IG:** 12 dyads.	Not reported	Intervention contents and dosage were the same as the one (Porter et al. [34]).	-Social workers; -Video conference.
Su et al. (2022) China [36]	-To explore feasibility and efficacy of CCST.	RCT	Gastric cancer (stages 3–4); Couples who presented cancer-related communication problem before intervention; **IG:** 44 dyads; **CG:** 43 dyads.	Not reported	**Reciprocal self-disclosure:** (1) express experience, thoughts and feelings sincerely through a cancer-related topic. **Partner responsiveness:** (1) reflectively listen to partner’s emotional disclosure, accept partner’s feelings and avoid giving advice quickly. **Relationship engagement:** (1) practice communication skills in each session; (2) try to make a cancer-related decision through communication skills; (3) write and read the letter of thanks for each other, review progress and discuss coping strategies for future cancer-related communication problems. **Relationship-compromising behaviors:** (1) point out couples’ unreasonable communication behaviors; five sessions (each 60 min) within five to seven weeks.	-Psychologist and nursing postgraduate; -In person.

**Table 2:** Outcomes of included studies. Symbols # and * could help us to distinguish whether the tools examine patients or spousal caregivers clearly.

Author (Year) Country [Reference]	Outcome Measurements (Measurement Intervals)	Program Evaluation Outcomes (Feasibility, Acceptability)	Intervention Effects (*p < 0.05* Indicates Statistical Significance)	
			Relationship Functioning	Individual Functioning
Fergus et al. (2014) Canada [23]	**Feasibility** -Recruitment and completion rate. **Acceptability** -Treatment Satisfaction Questionnaire (TSQ); At post-treatment. **Primary outcome measures** -Interview; At post-treatment.	**Feasibility** -Recruitment rate: 57.14%; completion rate: 62.5% of participants completed all modules; **Acceptability** -85% of couples satisfied with the program; the majority of couples regarded facilitator as helpful and effective; 61% of couples agreed that the website was easy to use; 55% of couples regarded the program as convenient; -Limitations: program disrupted couple’s usual routine, lacked profoundness and was impersonal.	**Within-group comparison of pre–post change** -The program opened couple’s communication channel, enhanced couple’s communication skill and improved meaningful cancer-related discussion; -Couples learned more relationship knowledge, perceived higher closeness with each other, and affirmed relationship strengths.	
Fergus et al. (2022) Canada [24]	**Feasibility** -Recruitment, retention and completion rate. **Primary outcome measures** -Relationship functioning: *positive dyadic coping*: Positive Dyadic Coping 19-item subscale of the Dyadic Coping Inventory; *relationship satisfaction (RS)*: Revised-Dyadic Adjustment Scale (DAS), Kansas Marital Satisfaction Survey; *self-perceived own dyadic coping*: The Breast Cancer and Relationship Measure; -Individual functioning: *anxiety and depression*: Hospital Anxiety and Depression Scale (HADS); At baseline, post-treatment and 3-month follow-up.	**Feasibility** -Recruitment rate: 89.3%; retention rate: 89% at post-treatment, 77% at 3-month follow-up; completion rate: all couples in IG completed at least five sessions.	**Within-group comparison of pre–post change** -Couples reported positive change in positive dyadic coping *(b = 2.40, p = 0.032, Cohen’s d = 0.24)* and perceived own dyadic coping *(b = 3.93, p = 0.04, Cohen’s d = 0.23)* in IG from baseline to post-treatment, but not from baseline to 3-month follow-up; -There was no significant change in couples’ RS regardless of treatment arms or follow-up periods.	**Within-group comparison of pre–post change** -Couples reported less anxiety from baseline to 3-month follow-up *(p = 0.03)*, but not from baseline to post-treatment in IG, while couples had reduced anxiety from baseline to post-treatment *(b = −0.58, p = 0.046)* and from baseline to 3-month follow-up *(b = −0.67, p = 0.022)* in CG; -There was no positive change in depression regardless of treatment arms or follow-up periods.
Fergus et al. (2022) Canada [25]	**Acceptability** -TSQ; open-ended questions to ask most and least helpful parts of the program, and feedback on the psychoeducational articles and videos. **Primary outcome measures** -Interview; After 1–2 weeks after post-treatment.	**Acceptability** -Couples satisfied with the program, while females reported higher satisfaction than males *(Cohen’s d = 0.42, p = 0.01)*; -The favorable parts were varied (e.g., “the variety of activity”, “the role playing” or “facing cancer as a unified front”), while limitations were lacking in-person contact, and novel learning.	**Within-group comparison of pre–post change** -Couples started to communicate with each other again, learned communication skills, spent quality time together, obtained insight into their relationship and perceived that they were “in this together”.	
Gremore et al. (2020) USA [26]	**Feasibility** -Recruitment, retention and completion rates. **Acceptability** -Client Satisfaction Questionnaire (CSQ); -Questions to assess most and least helpful aspects at post-treatment. **Primary outcome measures** -Relationship functioning: *RS*: DAS; *intimacy*: Miller Social Intimacy Scale (MSIS); -Individual functioning: *post-traumatic stress*: Impact of Events Scale (IES)—Revised; *anxiety*: Emotional Distress Scale–Anxiety; *depression*: The Center for Epidemiological Studies-Depression (CES-D); *patient quality of life (QOL)*: Functional Assessment of Cancer Therapy–Head and Neck #; *caregiver QOL*: Caregiver Quality of Life Index-Cancer *; Baseline, at post-treatment and 6-month follow-up.	**Feasibility** -Recruitment rate: 33.3%; retention rate: 80% for IG, 90% for CG; completion rate: 80% of IG participants and 90% of CG participants completed all sessions, 90% of participants in both IG and CG completed post-treatment, and 90% of IG participants and 80% of CG participants completed 6-month follow-up measures. **Acceptability** -CPs and their SCs were satisfied with the intervention, communicating about their feelings was helpful, but questionnaire measures were too long.	**Between-group comparisons of pre–post change** -CPs reported no more improvement in RS regardless of follow-up period, but more improvement in intimacy *(Cohen’s d = 0.42)* at post-treatment in IG than CG but no difference at 6-month follow-up; -SCs had more improvement in RS *(Cohen’s d = 0.53 and 0.47)* and intimacy *(Cohen’s d = 0.45 and 0.24)* at post-treatment and 6-month follow-up respectively in IG than CG.	**Between-group comparisons of pre- and post-change** -CPs had more anxiety (*t = 2.78, p < 0.05*) and depression (*t = 2.40, p < 0.05*) at baseline in IG than CG. -CPs reported more improvement in post-traumatic stress *(Cohen’s d = 0.22)* and QOL *(Cohen’s d = 0.48)* at 6-month follow-up but not at post-treatment, and more improvement in anxiety *(Cohen’s d = 0.65 and 1.17)* and depression *(Cohen’s d = 0.33 and 0.91)* at post-treatment and 6-month follow-up, respectively, in IG than CG; -SCs had more improvement in post-traumatic stress *(Cohen’s d = 0.88 and 0.87)*, anxiety *(Cohen’s d = 0.57 and 0.47)*, depression *(Cohen’s d = 0.45 and 0.61)* and QOL *(Cohen’s d = 0.22 and 0.31)* at post-treatment and 6-month follow-up, respectively, in IG than CG.
Manne et al. (2011) USA [27]	**Feasibility** -Recruitment, retention and completion rates. **Acceptability** -Questions for success of the intervention. **Primary outcome measures/Moderators** -Relationship functioning: *self-disclosure*: 3-item measure; *perceived partner disclosure*: 3-item measure; *perceived partner responsiveness*: 4-item measure; *mutual constructive communication*: The Mutual Constructive Communication subscale of the Communications Pattern Questionnaire (CPQ); *demand-withdraw communication*: The Demand-Withdraw subscale of the CPQ; *intimacy*: The Personal Assessment of Intimacy in Relationships; *RS*: DAS; -Individual functioning: *psychological distress*: The Psychological Distress scale of the Mental Health Inventory (MHI); *psychological well-being*: The Psychological Well-Being scale of MHI; cancer-*specific distress*: IES; *cancer concerns*: 10 self-report questions; Baseline, at eight weeks post-baseline.	**Feasibility** -Recruitment rate: 20.8%; retention rate: 94.6% for IG, 80.9% for CG; completion rate: 70.3% of participants completed all sessions. **Acceptability** -CPs and their SCs reported that sessions were quite successful.	**Between-group comparisons of pre- and-post change** -CPs reported higher scores of self-disclosure *(t = 3.50, p < 0.001)*, perceived partner disclosure *(t = 3.27, p = 0.002)*, and perceived partner responsiveness *(t = 3.26, p = 0.034)* in IG than CG when these variables were low before intervention; lower scores of self-disclosure *(t = −2.34, p = 0.022)* in IG than CG when the variable was high before intervention; -SCs had higher scores of mutual constructive communication *(t = 3.70, p < 0.001)*, intimacy *(t = 3.42, p = 0.001)* and RS *(t = 3.94, p < 0.001)* in IG than CG when these variables were low before intervention; lower scores of demand-withdraw communication *(t = −2.34, p = 0.023)*, intimacy *(t = −2.49, p = 0.015)* and RS *(t = −2.12, p = 0.038)* in IG than CG when these variables were high before intervention.	**Between-group comparisons of pre–post change** -CPs reported fewer cancer-related concerns *(t = −2.34, p = 0.022)* in IG than CG when the variable was high at baseline; -SCs had less cancer-specific distress *(t = −2.31, p = 0.024)* in IG than CG when the variable was high at baseline.
Manne et al. (2019) USA [33]	**Feasibility** -Recruitment, retention and completion rates. **Acceptability** -14 items assessed helpfulness and importance of the intervention; At three months post-baseline. **Primary outcome measures** -Relationship functioning: *RS*: DAS; -Individual functioning: *general psychological adjustment*: MHI-38; *depression*: The Patient Health Questionnaire-9 (PHQ-9); *cancer-specific distress*: IES; *cancer-related concerns*: 10 self-report questions; Baseline, at five weeks post-baseline, at three months post-baseline, and at six months post-baseline.	**Feasibility** -Recruitment rate: 15.2%; retention rate: 90% for IG, 78.9% for general health and wellness intervention, 85.2% for Usual Care group; complete rate: 86% of IG couples and 73.6% of CG couples attended five or six sessions; 89–92% CPs and 89–91% SCs completed homework; **Acceptability** -Satisfaction ratings were high for couples in both IG and CG.	**Within-group comparison of pre- and post-change** -CPs had no significant change in RS in three treatment arms over time; -SCs perceived improvement in RS in IG from baseline to five weeks post-baseline *(Cohen’s d = 0.22)*, but not at other follow-up periods.	**Within-group comparison of pre- and post-change** -CPs and SCs had significant improvement in general psychological adjustment, depression, cancer-specific distress, cancer concerns at varied follow-up periods regardless of treatment arms.
Mowll et al. (2015) Australia [28]	**Feasibility** -Recruitment and retention rates; **Acceptability** -Interviews; At two weeks post-treatment; **Primary outcome measures** -Interviews; At two weeks post-treatment.	**Feasibility** -Recruitment rate: 35.3%; retention rate: 75%; **Acceptability** -Most participants like the intervention and obtained some benefits, while one patient thought that psychologist did not discuss enough for discordance.	**Within-group comparison of pre- and post-change** -Male CPs and male SCs had more opportunities to talk; -The intervention promoted validated communication among couples which promoted closeness, mutual understanding and support.	
Porter et al. (2009) USA [29]	**Feasibility** -Recruitment and retention rates; **Primary outcome measures** -Relationship functioning: *intimacy*: MSIS; *relationship quality*: Quality of Marriage Index (QMI); -Individual functioning: *psychological distress*: Profile of Mood States-Short Form (POMS-SF); Baseline, at post-treatment; **Moderator** -*holding back*: 10 self-report items #; Baseline.	**Feasibility** -Recruitment rate: 24.9%; retention rate: 86.7%; completion rate: 83.1% of participants completed post-treatment assessment.	**Between-group comparisons of pre- and post-change** -CPs and SCs reported more improvement in intimacy and relationship quality in IG than CG only when CPs had high holding back from discussing cancer-related concerns before intervention.	**Between-group comparisons of pre- and post-change** -CPs and SCs reported no significant change in psychological distress between treatment arms.
Porter et al. (2012) USA [30]	**Primary outcome measures** -Relationship functioning: *intimacy*: MSIS; *relationship quality*: QMI; -Individual functioning: *psychological distress*: POMS-SF; *holding back*: 10 self-reported items #; **Process variables** -*Negative affect*: Negative Affect Subscale #; *observational ratings of patient expressiveness during the treatment sessions*: Self-Feeling Awareness Scale (SFAS) #; Baseline, at post-treatment and 8-week follow-up.	**Not reported**	**Between-group comparisons of pre- and post-change** -CPs and SCs reported more increase in intimacy and relationship quality at post-treatment and eight weeks follow-up in IG than CG when CPs had high holding back before intervention; -High level of CPs’ expression during sessions were related to promotion of couples’ intimacy and relationship quality at post-treatment.	**Between-group comparisons of pre- and post-change** -CPs and SCs reported no significant change in individual functioning between treatment arms; -High level of CPs’ negative affect immediately after each session were related to decline of couples’ psychological distress at post-treatment.
Porter et al. (2012) USA [31]	**Primary outcome measures** -Relationship function: *communication*: SFAS #, the Acceptance of Other Scale *, Post-session Self-Report Measures of Communication; At each post-session. **Moderators** *relationship quality*: QMI; *intimacy*: MSIS; *perceived partner avoidance and criticism*: 13-item scale; *disclosure and holding back*: ten domains of concern regarding gastrointestinal cancer; *psychological distress*: POMS-SF; Baseline.	**Not reported**	**Within-group comparison of pre–post change** -CPs rarely directly talked about their emotion; -SCs generally provided instrumental support rather than responding to the CPs’ emotion; -SCs were more possible to respond empathically when CPs were more expressive; -Empathic SCs were less likely to criticize their CPs; -CPs were rated as more expressive in IG when they reported lower relationship quality, higher partner avoidance, and higher holding back before intervention.	
Porter et al. (2017) USA [34]	**Feasibility** -Recruitment, retention and completion rates. **Acceptability** -CSQ; -Questions to assess the convenience of videoconference format, preference delivery format, and most and least helpful aspects of the intervention; At post-treatment. **Primary outcome measures** -Relationship functioning: *communication*: Problem Solving Communication subscale, Affective Communication subscale; *intimacy*: MSIS; *RS*: Revised-DAS; -Individual functioning: *cancer-related distress*: eight-item version of IES; *psychological growth*: Post-traumatic Growth Inventory (PTGI); *self-efficacy*: a standard self-efficacy scale; Baseline, at post-treatment.	**Feasibility** -Recruitment rate: 28%; retention rate: 80% for IG, 100% for CG; completion rate: 88% of participants completed all six sessions. **Acceptability** -Satisfaction ratings were high for CP and their SC in both treatment arms; -Participants reported highly satisfactory regarding videoconference format; rates of preference for formats of video conference, in person and telephone were 77%, 20% and 3%, respectively; most helpful aspect was listening skill.	**Between-group comparisons of pre–post change** -CPs reported more improvement in affective communication (*Cohen’s d = -0.35*), problem-solving communication (*Cohen’s d = −0.50*), intimacy (*Cohen’s d = 0.63*), and RS (*Cohen’s d = 0.30*) in IG than CG; -SCs perceived more improvement in RS (*Cohen’s d = 0.34*) in IG than CG, but not for communication and intimacy.	**Between-group comparisons of pre–post change** -CPs reported more improvement on the PTGI Personal Strength subscale (*Cohen’s d = 0.36*) in IG than CG, and greater increase in self-efficacy (*Cohen’s d = −0.22*) and PTGI New Possibilities (*Cohen’s d = −0.37*) in CG than IG; -SCs perceived more improvement on the PTGI relating to others (*Cohen’s d = 0.49*) in IG than CG, and more increase in self-efficacy (*Cohen’s d = −0.25*) in CG than IG. -No significant effect on cancer-related distress for CPs and SCs in IG than CG.
Porter et al. (2018) USA [35]	**Primary outcome measures** -Interviews; During the intervention.	**Not reported**	**Within-group comparison of pre- and post-change** -CPs and SCs discussed to attempt to maintain normal relationship with each other rather than being in the role of “patient” and “caregiver”; -CPs and SCs shared mutual understanding and addressed conflicts; -CPs desired to provide support to their SCs; -CPs and SCs discussed symptom-related emotional and practical considerations and made likely future treatment decisions; -CPs and SCs discussed death-related topics; -Couples highlighted importance of couple relationship for each other.	
Shields et al. (2004) USA [32]	**Feasibility** -Recruitment and completion rates. **Primary outcome measures** -Relationship functioning: *RS*: Revised-DAS; -Individual functioning: *mental health*: Mental Health Summary Score of The Medical Outcomes Study Short-Form (12 item scale) (SF-12); *cancer-related stress*: IES. Baseline, at post-treatment and 3-month follow-up.	**Feasibility** -Recruitment rate: 50%; completion rate: 82% of 2-session group, 73% of 1-session group, and 80% of comparison group completed time 3 assessment.	**Within-group comparisons of pre- and post-change** -CPs and SCs reported no visible change in RS in three study arms regardless of follow-up period.	**Within and between-group comparisons of pre- and post-change** -CPs had visible improvement of mental health and avoidance in 2-session group, but SCs had no visible change in each individual functioning in three study arms.
Su et al. (2022) China [36]	**Feasibility** -Recruitment and completion rates. **Primary outcome measures** -Relationship functioning: *communication*: Cancer-Related Communication Problem Scale (CRCP); *RS*: Relationship Assessment Scale (RAS); Baseline, at post-treatment.	**Feasibility** -Recruitment rate: 95.7% for IG, 93.5% for CG; completion rate: 90.9% of participants completed four to five sessions and 93.2% of participants completed home assignment.	**Within-group comparisons of pre- and post-change)** -CPs and SCs reported improvement in communication and RS in IG, but not in CG. **Between-group comparisons of pre- and post-change** -CPs and their SCs had more improvement in communication and RS at post-treatment in IG than CG.	

Notably, two trained reviewers independently extracted and synthesized data, and assessed the QR of the included studies. Discrepancies were resolved by the two reviewers through discussion until a consensus opinion was reached.

## 3. Results

### 3.1. Process of Study Selection

A total of 979 articles were found in eight databases and after additional manual searching, and 489 duplicates were excluded using EndNote 20. The titles and abstracts of the remaining 490 articles were reviewed based on inclusion and exclusion criteria. Of the 490 studies, 438 studies were excluded and 52 articles remained to be reviewed for the full-text component. Finally, 14 studies were included in this review for analysis. Figure 1 shows detailed information on the selection process. The most common reasons why studies were removed were that target population of studies were not couples, articles were not intervention studies or interventions did not focus on couple-based communication components.

### 3.2. Result of Quality Assessment

Table 3 illustrates the quality assessment of the involved articles. Three, seven, and four articles were rated as “strong”, “moderate” and “weak”, respectively. The rating of “weak” was mainly owing to a low recruitment rate of the target population, not using a blinding strategy and controlling confounders, and using tools without evidence of validity or reliability. Although the QR of the studies was varied, we included all of them because they basically completed their study aims and met the selection criteria of this review.

### 3.3. Characteristics of Intervention

Most intervention studies included in this review were carried out in western countries, including the USA (n = 9, 64.3%), Canada (n = 3, 21.4%) and Australia (n = 1, 7.1%), while one was from China (7.1%). The designs of the intervention studies were randomized controlled trial (RCT) (n = 8, 57.1%), single-group study (n = 5, 35.7%) and three-group study (n = 1, 7.1%). The single-group study included a pre–post single-arm study and interviews for participants from the treatment arm only.

### 3.4. Characteristics of Participants

Of the 14 articles involved in this review, 13 focused on a single type of cancer with varied stages, including gastrointestinal cancer (n = 5, 41.7%), breast cancer (n = 4, 28.6%), prostate cancer (n = 2, 14.3%), head and neck cancer (n = 1, 7.1%) and gastric cancer (n = 1, 7.1%), while one targeted any type of cancer with an advanced stage (n = 1, 7.1%). Notably, of the 13 articles testing a single type of cancer, seven focused on non-gender-specific cancer, while six targeted gender-specific cancer (e.g., breast or prostate cancer). The articles’ sample sizes differed, ranging from nine dyads to 237 dyads. Across the target population, 10 studies focused on participants regardless of the level of their relationship or individual functioning before the intervention [23,24,25,26,27,28,29,30,31,32], while four studies recruited those who had distress or cancer-related communication problems before the intervention [33,34,35,36]. In addition, ethnicity and education level of the majority of the participants in the included studies were Caucasian and college or higher, respectively.

### 3.5. Theoretical Framework of the Interventions

Six kinds of theoretical framework were used to instruct the research included in this review, including the developmental model of couple adaptation to illness [23,24,25], the theory of health relationship functioning [23,24,25], models of dyadic coping [23], systemic-constructive metatheory [24,25], social-cognitive processing theory [26] and RIM [26,27,33]. Among these theoretical frameworks, RIM was the most frequently demonstrated and was used to guide two kinds of intervention programs. Most included studies did not use a specific theoretical framework to guide their intervention, or some of those using a theoretical framework did not demonstrate how the framework guided their intervention in detail.

### 3.6. Intervention Content

In this review, interventions’ contents were summarized according to RIM, including four aspects: reciprocal self-disclosure, partner responsiveness, relationship engagement and relationship-compromising behaviors [11]. All 14 studies included self-disclosure contents, which primarily aimed to guide couples to disclose cancer-related experiences, thoughts and feelings. Of the 14 studies, 11 studies trained reciprocal self-disclosure [23,24,25,26,27,28,32,33,34,35,36], while the remaining three studies trained CPs’ unidirectional self-disclosure to their SCs [29,30,31]. Partner responsiveness contents were included in 13 studies [23,24,25,26,27,29,30,31,32,33,34,35,36], which mainly aimed to teach partners to become attentive listeners to help the other couple member feel understood and cared for. The main components included recording and discussing their own turning-toward and turning-away behaviors, patiently listening to their partner’s emotional disclosure, accepting and affirming their thoughts and feelings, responding with empathy and validation and avoiding giving advice quickly. Three studies only focused on the SCs’ unidirectional responsiveness to CPs [29,30,31]. Relationship engagement was defined as viewing cancer as a relational event and engaging in behaviors to maintain or strengthen the relationship in the context of cancer [11]. In this review, 11 studies included relationship engagement [23,24,25,26,27,28,32,33,34,35,36], comprising of practicing speaking and listening skills with cancer-related topics, improving problem-solving, highlighting individual and relationship strengths, enhancing a sense of “we-ness” in relation to cancer, co-constructing the relationship line about couples’ pivotal events/periods, writing and reading thanks letter to each other and identifying new goals for the future. With regard to relationship-compromising behaviors, pointing out or blocking unreasonable communication behaviors was reported [26,29,30,31,36].

### 3.7. Delivery Format and Intervention Dosage

#### 3.7.1. Delivery Format

The characteristics of the intervention deliverers were reported by 13 articles. Five articles’ intervention deliverers were psychologists [23,24,25,26,28], four were psychologists or social workers [29,30,31,33], two were social workers [34,35], one was therapists [27], and one was psychologists and nurses [36]. The delivery formats of the 14 studies varied, including in-person (n = 6, 42.9%) [27,28,29,30,31,36], online and telephone (n = 3, 21.4%) [23,24,25], video conference (n = 2, 14.3%) [34,35], in-person and telephone (n = 1, 7.1%) [33], in-person or video conference (n = 1, 7.1%) [26] and offline group interventions (n = 1, 7.1%) [32].

#### 3.7.2. Intervention Dosage

The number of modules/sessions in the 14 intervention studies was varied, ranging from one to six, with an average of 3.5. Of the 14 articles included in this review, 10 articles illustrated that each session generally lasted 60 to 90 min [26,27,29,30,31,32,33,34,35,36]. The length of each online module of three articles was chosen by the participants [23,24,25]. One study spent four hours in each session [28]. In addition, for the duration of the intervention, eight studies reported that the duration generally ranged from four to eight weeks, and the average value was six weeks [23,24,25,27,29,30,31,36]. Five articles did not mention the duration [26,32,33,34,35], while one study had only one session constructed during the first counselling session [28]. With regard to the follow-up periods, four studies had single follow-up timepoints, including eight weeks [30], three months [24,32] and six months after intervention [26], while another adopted multiple follow-up periods [33].

### 3.8. Feasibility and Acceptability

#### 3.8.1. Feasibility

The feasibility of the interventions was assessed in 10 studies. Eight studies reported generally low recruitment rates that were less than 60% [23,26,27,28,29,32,33,34], while two studies had relatively high recruitment rates above 60% (including one online intervention program conducted in Canada [24] and one in-person intervention conducted in China [36]). The reasons why participants refused to take part in the intervention mainly involved them being too busy, too far away, too sick, lacking interest or lacking need. It is worth noting that studies showed relatively high retention rates (ranging from 75% to 100%), and relatively high completion rates, which were demonstrated by the fact that 62.5–100% of participants completed most modules/sessions, 89–93.2% of participants completed relevant practice and 73–90% of participants completed outcome measurements.

#### 3.8.2. Acceptability

Seven studies examined the acceptability of their interventions. CPs and their SCs were highly satisfied with interventions [23,25,26,28,33,34] or viewed the intervention as quite successful [27]. Particularly, for one online intervention program, females reported higher satisfaction than males (Cohen’s d = 0.42, *p* = 0.01) [25]. The favorable aspects of programs for participants encompassed communicating their own feelings [26], listening skill [34] and “the variety of activity”, “the role playing” or “facing cancer as a unified front” [24]. In addition, limitations were also pointed out, including that the online intervention program lacked in-person contact [23,25], the questionnaire was too long [26] and the intervention lacked profoundness [23,28]. Approximately 60% of participants in the online intervention programs regarded website usage as convenient [23,25]. Another study reported that the rates of participants’ preferences for delivery formats were 77%, 20%, and 3% for video conference, in-person and telephone interventions, respectively [34].

### 3.9. Intervention Outcomes

All 14 studies measured relationship functioning, such as mutual communication, intimacy, and RS, and/or individual functioning, including anxiety, depression, psychological distress, cancer concerns, psychological adjustment and QOL for CPs and their SCs. The detailed information of intervention outcomes can be found in Table 2. In this review, the outcomes of relationship and individual functioning were separately analyzed according to within- and between-group comparisons of pre–post change.

#### 3.9.1. Effects on Relationship Functioning for Within-Group Comparisons of Pre–Post Change

Eight studies reported that CPs and/or their SCs experienced a significant improvement in their mutual communication [23,24,25,28,31,35,36], intimacy [23,25,28] and RS [23,25,33,35,36] in the intervention group (IG). For these significant outcomes, it was found that the interventions had a short-term (up to five weeks) impact on relationship functioning [33]. In addition, three studies reported that CPs and/or their SCs with distress or communication problems (e.g., holding back) before the intervention seemed to be more able to benefit from the interventions [33,35,36]. On the contrary, one online intervention [24] and another offline group intervention [32], which viewed RS as the primary outcome, did not improve the RS of either CPs or their SCs during multiple follow-up periods.

#### 3.9.2. Effects on Relationship Functioning for Between-Group Comparisons of Pre–Post Change

Gremore et al. [26] reported that CPs and their SCs, regardless of their level of relationship and individual functioning before the intervention, experienced more improvement in intimacy and RS in the IG than the control group (CG), even at six months follow-up. Another five studies showed that the intervention positively impacted relationship functioning only when CPs and/or their SCs had a low or negative functioning before the intervention [27,29,30,34,36]. Of the five studies, one demonstrated that CPs reported higher scores of self-disclosure (t = 3.50, *p* < 0.001), perceived partner disclosure (t = 3.27, *p* = 0.002) and perceived partner responsiveness (t = 3.26, *p* = 0.034), while their SCs had higher scores of mutual constructive communication (t = 3.70, *p* < 0.001), intimacy (t = 3.42, *p* = 0.001) and RS (t = 3.94, *p* < 0.001) in the IG than the CG only when these variables were low before the intervention [27]. The remaining four studies illustrated that CPs and/or their SCs obtained a greater increase in communication, intimacy, and/or RS in IG than CG only when at least one of them had communication problems (e.g., holding back) before intervention [29,30,34,36]. Notably, the offline group intervention had no any significantly different influence on couples’ RS between treatment terms regardless of at post-treatment or three months follow-up [32]. Unexpectedly, Manne, et al. [27] found that the intervention may diminish level of self-disclosure of CPs, and intimacy and RS of their SCs when these variables were high before the intervention.

#### 3.9.3. Effects on Individual Functioning for Within-Group Comparisons of Pre–Post Change

Two studies explored the effect of interventions on individual functioning in within-group comparisons of pre–post change [24,33]. One online intervention program reported that couples perceived less anxiety at follow-up periods in both the IG and the CG [24]. The other study, which focused on participants with distress before the intervention, reported that CPs and their SCs experienced an improvement in psychological distress, depression, cancer concerns and psychological adjustment during varied follow-up periods regardless of treatment arms [33].

#### 3.9.4. Effects on Individual Functioning for Between-Group Comparisons of Pre–Post Change

Seven studies investigated the different influence of treatment arms on psychological adaptation and/or QOL. Greater improvements in post-traumatic stress, anxiety, depression and psychological adjustment at both post-treatment and six-month follow-up were reported by CPs and their SCs in the IG than the CG [26]. Moreover, Manne et al. [27] demonstrated that couples had fewer cancer-related concerns (t = −2.34, *p* = 0.022) or cancer-specific distress (t = −2.31, *p* = 0.024) in the IG than the CG, but only when these variables were high before the intervention. The offline group intervention found that CPs had more visible improvement in psychological adjustment in the two-session group than the other two treatment arms, but SCs did not [32]. In addition, Porter et al. [34] illustrated that couples seemed to gain more improvement in post-traumatic growth and self-efficacy in the CG than the IG, when the CG aimed to provide cancer-related information over six sessions [34]. Two studies that were designed to promote CPs’ unidirectional self-disclosure to their SCs reported no significant change in the psychological distress of couples between treatment arms, regardless of follow-up periods [29,30]. As for QOL, CPs had a greater increase in QOL at six-month follow-up (Cohen’s d = 0.48), while their SCs experienced more improvement at post-treatment (Cohen’s d = 0.22) and six-month follow-up (Cohen’s d = 0.31) in the IG than the CG.

## 4. Discussion

A total of 14 eligible articles were included in this review according to the inclusion and exclusion criteria. Through extracting and synthesizing characteristics and outcomes of these included studies, we have demonstrated the approaches and contents used in couple-based communication interventions, their feasibility and acceptability, and their effect on CP–SC dyads’ psychosocial adaptation to cancer. To synoptically discuss the results of these included studies, we have arranged the construction of the Discussion section as four “Ws” (including Who, What, How, and When), efficacy, recommendations and limitations to provide some enlightenment for future research.

### 4.1. Who?—Choosing the Target Population

For CPs and their SCs, the diagnosis of specific types of cancer would induce specific communication needs. For example, CPs with colorectal cancer would like to communicate about ostomy-related issues and changed bowel function with their SCs [37]. There are different communication topics among couples during different cancer stages. Facing early diagnosis, CPs and their SCs may be more concerned with the treatment effect, a healthy diet and physical exercise. Meanwhile, for advanced cancer stages, there will be more difficult topics, such as making the CPs comfortable rather than cured and anticipatory grief of losing loved partners [38]. In addition, half of the articles included in this review targeted a population with gender-specific cancer (breast or prostate cancer). Gender may act as a possible factor impacting adjustment outcomes for CPs and SCs. For instance, in the context of colorectal cancer, studies reported that female CPs and female SCs usually experienced more anxiety, depression, fear of cancer recurrence [39,40,41,42] and less marital satisfaction [43] than their male counterparts. Although a systematic review and meta-analysis conducted by Hagedoorn et al. found that individual levels of distress were attributed more to gender compared with role (CP or SC) [44], focusing on participants with gender-specific cancer still makes it difficult for researchers to distinguish whether gender or role has an impact on adjustment outcomes [45]. In addition, according to intervention outcomes in this review, interventions seemed to be more beneficial for participants who experienced distress or communication problems before the intervention. Notably, one study led by Manne et al. reported that a couple-based communication intervention may reduce participants’ level of self-disclosure, intimacy and RS when they had relatively high levels of relationship functioning before the intervention [27]. More research is warranted to explore whether it is necessary to assess couples’ relationship or individual functioning before the intervention and select those with distress or communication problems as participants.

### 4.2. What?—Communication Topics

Communication topics mentioned in the included studies consisted of cancer-related experience, thoughts, feelings and relationship issues. Encouraging cancer-related emotional disclosure among CP–SC dyads is a common recommendation in couple-based interventions [46]. Badr reported that there is great variability in topics when couples talk about cancer [17]. Except for emotional disclosure, health-related topics (e.g., symptom management, treatment issues, daily care and prognosis) and relationship topics (e.g., role change, marital relationship and social/family issues) also need to be given more attention [17,47]. For example, talking about symptom management/treatment issues possibly helps couples reassert a sense of control over their cancer situation [17], talking about daily care (e.g., healthy diet and exercise) may promote couples going back to a routine life, while discussing relationship topics may activate support resources from the relationship network [11]. Therefore, it is necessary to emphasize the importance of varied topics for CP–SC dyads’ psychosocial adaptation to cancer, including cancer-related emotional disclosure, healthy issues and relationship topics.

### 4.3. How?—Communication Methods

Teaching couples how to effectively communicate with each other was the critical content in these interventions in this review, mainly comprising of improving beneficial reciprocal self-disclosure, partner responsiveness and relationship engagement. In this review, unidirectional self-disclosure and partner responsiveness may have had limitations because it overlooked the process of SCs’ self-disclosure and CPs’ responsiveness. Manne et al. [48] reported that, regardless of role (CP or SC), self-disclosure and partner disclosure positively promoted the perceived partner responsiveness, and further improved their intimacy, which may be attributed to reciprocal self-disclosure and partner responsiveness being important for improving a couple’s members’ sense of feeling understood, validated and cared for [11]. In addition, distinguishing and declining relationship-compromising behaviors are similarly important because of their consistent association with negative outcomes [17]. As for ways of expression, except for oral discussion, this review showed that written expression was also used and was effective. With regard to communication channels, some participants regarded themselves as “face-to-face kind of person” [25], while other participants preferred using social media to communicate [47]. Therefore, it is more reasonable to provide varied communication channels to be chosen by participants.

### 4.4. When?—Communication Timing

With regard to intervention dosage, in general, the included studies implemented interventions in 3–4 modules/sessions (each 60–90 min in length) within six weeks, and had up to six-month-long follow-up periods. For some participants with specific situations, such as a low capability to understand, more need to communicate and disruption due to treatment, fittingly lengthening the intervention dosage is necessary. As for when to start the coupled-based communication interventions, previous studies suggested that it is better to start before some important treatment/life timepoints, such as before making a decision about treatment, surgery, the transition to survivorship and end-of-life care [11,47].

### 4.5. Exact Efficacy of Couple-Based Communication Interventions

Generally, the feasibility and acceptability of existing couple-based communication interventions are acceptable. The relatively low recruitment rates may not only have been due to the difficulty of conducting the psychological program, but also obstruction of recruiting CPs and their SCs simultaneously. The relatively high retention and completion rates may suggest that the interventions could greatly meet participants’ communication needs, which, in turn, supports the view that it is necessary to carry out this kind of intervention to help couples adjust to cancer better. The effect sizes of interventions were identified as small (Cohen’s d = 0.20–0.30), medium (Cohen’s d = 0.30–0.60) and large (Cohen’s d > 0.80) [26]. In this review, two studies reported small effect sizes, ranging from 0.22 to 0.30 [24,33], which were consistent with the result of a review conducted by Badr and Krebs [19]. Another two studies showed medium [34] to large effect sizes [26], ranging from 0.31 to 1.17. The relatively high effect sizes may be attributed to the clear and singular purpose of couple-based communication interventions, or the fact that they focused on participants with distress or communication problems before the intervention. It is still difficult to conclude what the reasons were for the medium to large effect sizes of interventions due to insufficient studies.

In addition, Badr, et al. suggested that researchers should move beyond a “one size fits all” approach [48,49], explore nuances of couples’ communication and develop more efficacious interventions for promoting CP–SC dyads’ psychosocial adaptation to cancer [17]. For example, different interactions of gender and role seem to result in varied communication performance during dyadic coping with cancer. As for female patients with male partners, male partners usually initiated cancer-related communication (e.g., treatment options), but most of them avoided communicating emotional reactions [50]. Lyons et al. [51] found that female patients experienced less depression when their male partners engaged in a lower level of protective buffering (e.g., hiding worries or waving patients’ worries aside). When it came to male patients with female partners, both of them tended to deny, avoid, refuse and hold back cancer-related discussion, which may be because they wanted to protect each other, or they assumed their partner’s situations, thoughts and feelings rather than assessing and resolving their partner’s actual emotional problems [50]. Although male patients perceived less depression when their female partners engaged in a high level of protective buffering [51], female partners’ desire to gain more emotional reaction and information from male patients needed to be given more attention [50]. According to social role theory [52], the gender difference of communication performance may be attributed to gender stereotypes, that is, men are good at instrumental behaviors while women prefer expressive behaviors. Exploring reasons or motivations for specific communication performance induced by role and gender would be helpful for researchers to better understand this specificity and provide CP–SC dyads with more personalized communication support.

### 4.6. Recommendations for Future Research

According to the characteristics and outcomes of intervention studies included in this review, we give the following recommendations in the hope of helping future relevant interventions:

(1) Conducted country: 93% of couple-based communication interventions were carried out in western countries, which reminds us that future interventions should be investigated in other regions, such as Asia;
(2) Target population: more research is needed to explore the necessity of screening distress or communication difficulties of CPs and/or SCs before the intervention;
(3) Study design: more longitudinal RCTs with large enough sample sizes are needed to explore the efficacy of couple-based communication interventions;
(4) Theoretical framework: a detailed combination of the theoretical framework and intervention design adopted should be reported in order to support future replicative studies;
(5) Intervention contents: focusing on varied topics, teaching couples to improve mutual communication behaviors, activating couples’ relationship resources and considering specificity of gender and role during the intervention may promote the efficacy of couple-based communication interventions;
(6) Intervention delivery: to meet the CP–SC dyads’ different preferences for delivery format, a combination of in-person and web-based intervention delivery is recommended to be adopted.

### 4.7. Limitations of this Review

It is necessary to point out that there are several limitations in this review. First, only studies published in English or Chinese were searched for and included in this review. Potential studies in other languages or published forms (e.g., conference proceedings, dissertation or editorials) may have been overlooked. Second, the heterogeneity of the included studies, such as differences in the type and stage of cancer studied, the study design, and the varied measurement tools used, may have impacted the generalization of outcomes. Third, we should be cautious to interpret the effect sizes of the interventions because they had small sample sizes and different baseline functioning. In sum, more research is needed to improve understanding and develop more efficacious couple-based communication interventions.

## 5. Conclusions

CP–SC dyads may experience communication difficulties while coping with cancer. Studies included in this review reported that a couple-based communication intervention improved CP–SC dyads’ relationships and individual functioning, that is, to improve couples’ psychosocial adaptation to cancer. More research is warranted to understand and develop more efficacious couple-based communication interventions, such as exploring the impact of the interaction of gender and role on dyads’ mutual communication behaviors.

## Figures and Tables

**Table 3:** Quality assessment of the included studies (n = 14).

Author; (Year); Country [Reference Number]	Selection Bias	Design	Confounders	Blinding	Data Collection	Dropouts	Quality Rating
Fergus et al. (2014) Canada [23]	W	M	W	W	W	M	**W**
Fergus et al. (2022) Canada [24]	S	S	S	M	M	S	**S**
Fergus et al. (2022) Canada [25]	S	M	M	M	W	S	**M**
Gremore et al. (2020) USA [26]	W	S	M	S	M	S	**M**
Manne et al. (2011) USA [27]	W	S	S	M	S	S	**M**
Manne et al. (2019) USA [33]	W	S	S	M	S	S	**M**
Mowll et al. (2015) Australia [28]	W	M	W	W	W	S	**W**
Porter et al. (2009) USA [29]	W	S	S	S	M	S	**M**
Porter et al. (2012) USA [30]	W	S	S	M	M	M	**M**
Porter et al. (2012) USA [31]	M	M	S	S	M	S	**S**
Porter et al. (2017) USA [34]	W	S	M	S	M	S	**M**
Porter et al. (2018) USA [35]	M	M	W	M	W	S	**W**
Shields et al. (2004) USA [32]	W	M	W	W	M	M	**W**
Su et al. (2022) China [36]	S	S	S	M	M	S	**S**

Selection bias: Strong—very likely to be representative of the target population and greater than 80% participation rate; Moderate—somewhat likely to be representative of the target population and 60–79% participation rate; Weak—all other responses or not stated. Design: Strong—RCT and CCT; Moderate—cohort analytic, case–control, cohort or an interrupted time series; Weak—all other designs or design not stated. Confounders: Strong—controlled for at least 80% of confounders; Moderate—controlled for 60–79% of confounders; Weak—confounders not controlled for, or not stated. Blinding: Strong—blinding of outcome assessor and study participants to intervention status and/or research question; Moderate—blinding of either outcome assessor or study participants; Weak—outcome assessor and study participants were aware of intervention status and/or research question. Data collection methods: Strong—tools were valid and reliable; Moderate—tools were valid but reliability not described; Weak—no evidence of validity or reliability. Withdrawals and dropouts: Strong—follow-up rate of >80% of participants; Moderate—follow-up rate of 60–79% of participants; Weak—follow-up rate of <60% of participants or withdrawals and dropouts not described. Quality rating: S: strong; M: moderate; W: weak. Strong: If a study had no weak ratings and at least four strong ratings, then it was considered strong; Moderate: If the study had fewer than four strong ratings and one weak rating, it was rated moderate; Weak: If a study had two or more weak ratings, it was considered weak.

## Data Availability

All data used in this study are completely published online.
